# Exploration of Work and Health Disparities among Black Women Employed in Poultry Processing in the Rural South

**DOI:** 10.1289/ehp.7912

**Published:** 2005-07-18

**Authors:** Hester J. Lipscomb, Robin Argue, Mary Anne McDonald, John M. Dement, Carol A. Epling, Tamara James, Steve Wing, Dana Loomis

**Affiliations:** 1 Division of Occupational and Environmental Medicine, Department of Community and Family Medicine, Duke University Medical Center, Durham, North Carolina, USA; 2 Occupational and Environmental Safety, Duke University, Durham, North Carolina, USA; 3 Department of Epidemiology, School of Public Health, University of North Carolina, Chapel Hill, North Carolina, USA; 4 Department of Environmental Sciences, School of Public Health, University of North Carolina, Chapel Hill, North Carolina

**Keywords:** African American women, black women, community-based participatory research, health disparities, musculoskeletal disorder

## Abstract

We describe an ongoing collaboration that developed as academic investigators responded to a specific request from community members to document health effects on black women of employment in poultry-processing plants in rural North Carolina. Primary outcomes of interest are upper extremity musculoskeletal disorders and function as well as quality of life. Because of concerns of community women and the history of poor labor relations, we decided to conduct this longitudinal study in a manner that did not require involvement of the employer. To provide more detailed insights into the effects of this type of employment, the epidemiologic analyses are supplemented by ethnographic interviews. The resulting approach requires community collaboration. Community-based staff, as paid members of the research team, manage the local project office, recruit and retain participants, conduct interviews, coordinate physical assessments, and participate in outreach. Other community members assisted in the design of the data collection tools and the recruitment of longitudinal study participants and took part in the ethnographic component of the study. This presentation provides an example of one model through which academic researchers and community members can work together productively under challenging circumstances. Notable accomplishments include the recruitment and retention of a cohort of low-income rural black women, often considered hard to reach in research studies. This community-based project includes a number of elements associated with community-based participatory research.

Classic epidemiologic studies and historical research document that in the past, blacks in particular were openly selected for unpleasant, “dirty” jobs regarded as unsuitable for other workers (Baron 1983; Cherniak 1986; Lloyd 1971; Taylor and Murray 2000). Historically, employers have also sought to reduce labor costs by hiring workers from less-advantaged groups—notably racial minorities, immigrants, women, and children—who are perceived to be willing to accept lower pay and poorer working conditions and to be less likely to organize (Green 1983; Levine 1989).

The U.S. labor force continues to be segregated by race and gender (Darrity 2003; Thompson et al. 2005; Tomaskovic-Devey 1993). Blacks are employed in hazardous occupations more frequently than whites, and black men experience higher occupational fatality rates than white men employed in the same jobs (Loomis and Richardson 1998). Compared with other women, African American women have higher rates of nonfatal occupational injuries treated in emergency departments in the United States (Chen and Hendricks 2001), with differences in employment by racial group suggesting explanations for this pattern. Recent evidence indicates that working conditions may be particularly dangerous for nonwhite workers in the Southern United States (Richardson et al. 2004).

Inequities in rights to medical care and wage replacement under workers’ compensation are also present. Claim rejection for carpal tunnel syndrome has been reported to be strongly related to ethnicity and socioeconomic status, with claims by nonwhites, low-wage earners, and union members more likely to be challenged. Although more than 96% of the adjudicated cases in this report were eventually approved, the mean time involved in the process was over 1 year (Herbert et al. 1999). In Canada a recent report indicates that women are less likely to have contested claims for musculoskeletal disorders (MSDs) accepted by an appeals tribunal than their male counterparts (Lippel 2003). The mislabeling and early mismanagement of these disorders may affect their clinical course (Andersson et al. 1995; Buckle 1997; Oschner et al. 1998) and may have adverse consequences for women who balance multiple roles at home and work.

The Safety and Health of Working Women (SHOWW) project is a collaboration of academic researchers and community representatives in rural northeastern North Carolina designed to explore occupational roots of health disparities. Both the tradition of hiring the less advantaged in less desirable and often more dangerous jobs and the failure to acknowledge their occupational health concerns are at the core of this research. We present the conceptual framework for the project and describe the development of the collaboration as an example of one model through which academic investigators and community members can work effectively under challenging circumstances.

## Project History and Setting

In 1999 women working for a small nonprofit advocacy organization sought an academic partner to document health effects of employment on black women working in poultry-processing plants. Several of these women had experience with community and academic collaboration surrounding environmental health issues (Wing et al. 1996). Upper extremity MSDs and acute injuries were viewed as the primary, but not the only, outcomes contributing to poor health among these working women. Women maintained that management and health care providers often attributed their musculoskeletal complaints to obesity, child-care responsibilities, or conditions existing prior to their employment.

The study area, which includes five counties located in northeastern North Carolina, is poor and sparsely populated. Fifty-five percent of the five-county population is black, compared with 22% in North Carolina and 12% throughout the United States (U.S. Census Bureau 2004a). This black majority is a legacy of the large slave population of the antebellum plantation system (Powell 1988). The area is characterized by poor health indices, including high age-, race-, sex-adjusted total mortality, low birth weight, and high infant and fetal mortality. These five counties rank in the top 15% in the state for years of potential life lost, with two of the counties being the highest in the state (Center for Health Services Research and Development 2003). In North Carolina 78% graduate from high school (80% in the United States), whereas in the study area, only 66% graduate. Nearly one-third of the population lives below the poverty level (U.S. Census Bureau 2004b, 2004c, 2004d, 2004e, 2004f, 2004g). Although North Carolina has enjoyed rapid economic expansion and high population growth during the last 20 years, the study area has remained economically underdeveloped. Poultry processing is the largest employer in the area, providing yearly salaries in the range of $17,000.

The development of the modern poultry industry and the economy of the rural South are closely connected. The production, processing, and marketing of poultry products have undergone a massive transformation. In 1960, 300 commercial suppliers and a larger number of family farms supplied chickens for the retail market. By 1990, fewer than 50 firms remained, and the top five among them supplied over a third of the market. Both production and processing of poultry are concentrated in the rural South (Fink 2003; Griffith 1993; Hall 1989).

Modern poultry-processing plants are highly organized industrial structures for slaughter, disassembly, and packaging of birds (Campbell 1999). Rapid line speed and extreme division of labor characterize the assembly-line work. The concentration of this low-wage industry in depressed rural areas and the employment of large numbers of black and Hispanic women help producers keep costs low (Fink 2003; Griffith 1993; Hall 1989) contributing to an environment that fosters disparities in working conditions and health.

The poultry plants in our study area have a history of occupational health and safety problems. In 1989, N.C. Occupational Safety and Health Administration (OSHA) inspectors cited the two plants in the area for serious repetitive motion problems with some jobs documented to require over 10,000 repetitions per shift (North Carolina Department of Labor 1989). After the citations the National Institute for Occupational Safety and Health (NIOSH) conducted Health Hazard Evaluations (HHE) in these plants that processed over 400,000 birds daily (Kiken et al. 1990). At the two plants, 36% and 20%, respectively, of employees who participated in the NIOSH evaluation had work-related cumulative trauma disorders in the last year, as determined by questionnaire alone; 20% and 8%, respectively, had current work-related disorders based on both questionnaire responses and physical exam. At both plants the high-exposure group included larger percentages of women, blacks, and younger people. At one plant, all high-exposure participants were black. The HHE clearly demonstrated risk. However, annual turnover rates in both plants were high (50–70%), and because workers with painful disorders tend to leave employment, this cross-sectional study of disease prevalence may underestimate the magnitude of morbidity because of “survivor bias.”

## Conceptual Framework for the Project

Musculoskeletal disorders are a frequent reason for seeking medical care and common causes of chronic health problems and long-term disability (Badley et al. 1994). Despite a body of literature linking occupational exposures such as repetitiveness, force requirements, posture, vibration, and lifting with MSDs, especially at high levels of exposure (NIOSH 1997), the majority of studies add little to understanding natural history (including latency and cumulative exposure) or resulting impairment and disability because of their cross-sectional nature.

**Figure 1:**
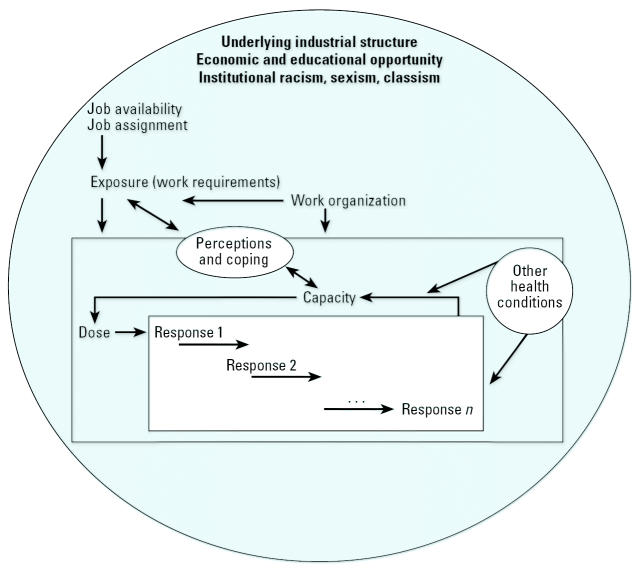
Conceptual framework for the study of MSDs among rural women employed in poultry processing. Figure modified from Armstrong et al. (1993).

From this background, through discussions with the women who requested the research partnership, interviews with workers, and review of historical information on the poultry industry and the geographic area, we developed the conceptual framework illustrated in Figure 1. The framework draws heavily on a conceptual model of work-related neck and upper-limb MSDs described by Armstrong et al. (1993) that incorporates relationships among exposure, dose, response, and worker capacity.

Exposure refers to the external factors, or work requirements such as repetition, force, and postures, that produce the internal dose (tissue loads, metabolic demands) on the worker. Dose refers to factors that disturb the individual mechanically, physiologically, or psychologically. Response includes the changes that occur in the individual in response to dose. Capacity, physical or psychological, refers to the ability of the individual to resist destabilization and is influenced by prior dose as well as other factors including health conditions*.*

The model posits that response at one level can affect dose at another level, and that the relationship between dose and response can be altered by previous dose. For example, repeated and prolonged exertions can result in desirable adaptation, as in a training effect on muscle or in undesirable reduced capacity, when a muscle is fatigued repeatedly without sufficient time to recover. Besides the direct effect of dose on tissue, the response of one tissue can affect another tissue. Connective tissue can thicken as it adapts to mechanical stress, and the thickening can lead to pressure on neural structures. Changes in upper-extremity function may represent pain and/or early tissue changes that, in turn, affect the individual’s capacity to withstand additional dose.

In addition to the physical job requirements, worker capacity, skill, and the social and physical organization of the work environment may influence the development and/or expression of MSDs. Yet these relationships are highly complex and contextual (Hagberg 1992). For example, work longevity, job stress, and organizational factors such as levels of psychological demand and control (Karasek et al. 1998) are potentially important modifiers of work exposures. In the case of MSDs, the control a person has over how she works might influence her work speed, breaks, and voluntary task rotation. Individuals with more experience may learn to use tools more efficiently with less force (Dempsey and McGorry 2004).

We also view perceptions and coping mechanisms as potentially significant modifiers of exposure and individual capacity. A worker with impaired upper extremity function who alters the manner in which she does the work changes her subsequent exposures. In a poultry plant, a woman’s assertive behaviors might alter physical exposures by securing job rotation or sharper tools; conversely, situations could arise where complicity might gain favor from superiors. James et al. (1987) have described “John Henryism,” a strong personality predisposition to cope actively with psychosocial environmental stressors. The scale developed to measure this attribute includes Likert-scaled items such as “When things don’t go the way I want them to, that just makes me work even harder.” The potential for high active coping among disadvantaged women may be maladaptive, leading them to experience greater damage through harder work and greater internalized stress. Poultry-processing workers represent the lower end of the socioeconomic distribution among workers, and we are interested in how their level of socioeconomic disadvantage influences decisions about work. These complex relationships of workplace exposures with adaptive and pathological responses may be most appropriately considered as interdependent rather than independent effects, as indicated by the double-headed arrows in Figure 1.

Obviously, both job availability and assignment determine individual exposures. However, to emphasize the importance of context, the sphere in which the model sits represents the underlying industrial structure. Unstated policies, such as institutional racism, sexism, and classism, as well as stated ones, such as economic development plans, cannot be measured through a focus on individuals as independent actors. In seeking to understand contributions of work to health disparities, it is important to understand what happens in workplaces and how workers respond, but also how workplaces come to be in certain communities and what employment alternatives workers may or may not have.

## Research Design and Methods

To address the concerns of women in the community and to improve cross-sectional investigations, we are conducting a longitudinal study of a volunteer cohort of women employed in poultry processing. Based on community concerns and the history of the industry’s poor labor relationships (Fink 2003; Griffith 1993; Human Rights Watch 2000), we decided to conduct the study in a manner that did not require the cooperation of the employer. This critical decision influenced the research methods and, particularly in a nonunion environment such as this, necessitated community involvement.

Volunteers participate in serial interviews and physical exams conducted at 3- to 6-month intervals over a maximum of 3 years. Community staff recruited participants over a 23-month period providing, by design, variable lengths of follow-up time and, consequently, variable cumulative occupational exposures. Although we initially limited recruitment to new hires to the industry, we later included longer-term employees to increase the overall efficiency of the study.

Table 1 outlines key variables. The primary outcomes of interest ( “responses” in our conceptual framework) are upper extremity musculoskeletal symptoms and disorders. Disorders are defined by a constellation of reported symptoms and signs identified by standardized physical exams performed by study nurses. The longitudinal design of our study allows us to explore relationships among health outcomes, tenure in the plant, exposure differences, and coping strategies. We also will be able to investigate upper extremity function, health-related quality of life, and depressive symptoms as outcomes, as well as subsequent modifiers of the relationships among our primary outcomes and exposures.

We developed a multidimensional strategy of exposure assessment that will result in two streams of data for analyses: one based on group-level exposure assignment by department and job, and the second derived from individual-level self-report of exposure. The process began with in-depth, semistructured interviews with 37 workers from different departments and jobs. We are using the information from these workers, combined with general knowledge of the poultry-processing industry (OSHA 2004), to construct an industry-specific job exposure matrix (JEM) (Bouyer and Hemon 1993; Kauppinen et al. 1998; Le et al. 1998). The matrix will be used to assign levels of exposure for key variables such as repetition, force, and joint posture by department and job. JEMs have been used effectively to combine observational or direct exposure measurements with past work histories to derive a measure of overall exposure for both surveillance and etiologic research. They provide a global evaluation of a job category that can be used to estimate exposures by job, or task, with cumulative exposures based on length of employment. Relevant to this project, JEMs facilitate exposure assessment when the workplace is not accessible to the researcher (Siemiatycki et al. 1981).

In addition, information from these key informant interviews guided the development of a self-report exposure assessment tool. The aggregated self-report data from women in the same jobs will be used in the JEM to help assign categories of exposure. The potential importance of individual behaviors, even in this assembly-line work, emerged in the analyses of the interview data. Long-term workers described behaviors that could potentially alter physical exposures, such as demanding sharper tools, taking unauthorized breaks, or refusing part of a job rotation or task. These are included in the assertiveness measure referenced in Table 1, and they may help us understand differences in the group-level exposures based on the JEM and individual reports of exposure.

The final component of our research design involves the documentation of workers’ life histories through in-depth, ethnographic interviews that explore how their work affects their lives and how they make decisions about their employment. These interviews, separate from those conducted early in the project to inform the exposure assessment, are designed to provide a broader understanding of quality of life and a view of more dimensions of the poultry workers’ lives. We are interviewing women who have worked in the industry for variable periods of time, as well as some who are no longer employed in poultry processing. These interviews are typically conducted in several sessions (up to three) and over 1–3 years. We intentionally tried to find women who represented ranges of age, length of employment in the plant, satisfaction with work, and injury and disability, and who lived throughout the five-county study area. We relied on SHOWW community–based staff to recommend women to be interviewed. They suggested women they knew through social networks or kinship, as well as women who were enrolled in the longitudinal study, who seemed open and forthcoming during the staff-administered study questionnaire sessions. This was not intended to be a random sample; when using ethnographic interviews, the goal is to find people who are knowledgeable, representative of the population of interest, and willing to talk (Fetterman 1998; Patton 2002; Ulin et al. 2005). Twenty-two different women were interviewed in 35 sessions. The ethnographic interviews covered life history, employment choices, family responsibility, hopes and fears, role of the church in their lives, social relationships within the plant, and many other topics.

## Collaboration of Community and Academic Research Team

Our research design not only affords opportunities for, but requires, collaboration. Early in the project the community nonprofit agency that was our original partner was unable to meet the obligations of the research project. Since then, the project has not had an independent community partner, but rather community members who serve as paid staff at a project office located in the study area. With this arrangement the staff serving as community representatives are not agents of a community organization but work directly with the research team as university employees. Key elements of this collaboration and support provided to community staff are presented in Table 2.

The five members of the community-based team are all black women raised in northeastern North Carolina and range from 19 to 60 years of age. Although demographic similarities exist among the staff, their life and work experiences vary considerably; they bring diversity of expertise and skill to the study. All five women obtained a high school diploma; two continued on for an associate degree. Their collective work backgrounds include positions in poultry processing (nearly 30 years) and sewing factories (5 years); grocery stores (manager for 12 years and clerk for 1 year); nursing homes (2 years); beauty salons (3 years); and community-based service organizations (20 years). These women are our main connection to the community and actively participate in shaping the research.

The community-based staff had no experience with a participatory or collaborative work-place. To augment their initial enthusiasm, to ensure that they would see themselves as integral to the project, and to foster equitable participation, we designed the project training using the elements of participatory learning (Arnold et al. 1991; Wallerstein and Rubenstein 1993). Key tenets of this method are equality among leaders and learners, and reciprocal learning. Participatory training methods enabled us to build on their existing knowledge of the poultry industry and of their communities. By creating a training where participants’ knowledge was valued, we strived to set the tone for collaboration based on equality and mutual respect.

These women work independently in an office 2.5 hr away from the university team, which gives them autonomy and responsibility. Community staff recruit participants and schedule their initial and follow-up interviews and examination appointments. They provide transportation, offer childcare and activities (crayons, books, or toys) for those who need to bring their children, and offer to conduct interviews in workers’ homes to facilitate participation. Data are collected through in-person interviews conducted by the staff using flip charts. Workers are able to view options to closed-ended questions as well as see visual cues, allowing participation by individuals who might otherwise have difficulty completing a lengthy questionnaire.

In addition to identifying avenues for outreach in their community and attending various community events or meetings to discuss the project, the community staff provide valuable outreach to the academic community through participation in university classes in occupational epidemiology, community-driven research, and political science, providing important community perspectives to students and faculty.

The academic project manager meets weekly with staff in the community. The meetings serve several purposes including information sharing, timely quality control of data, and problem solving. This regular forum promotes communication and allows dedicated time when input from staff is actively solicited and decisions can be made collectively.

Although the academic team made the decision to conduct a longitudinal study, that decision was influenced by expressed concerns over the inability to document work-related disorders among community women, and the women agreed with the need for and logic of a longitudinal design. The community staff have remained routinely involved in design decisions. They influenced the decision to incorporate ethnographic methods by requesting ways in which interested workers with longer tenure in the plant or former workers could be involved. The community staff also felt that longer-term workers should have the opportunity to participate in the longitudinal study and that their involvement would facilitate recruitment of new hires to the industry.

Input from current and former workers in the plants has also been critical in developing a self-report tool to capture personal exposure information. They provided information on potential sources of exposure variability that could be overlooked even in ergonomic assessments where investigators had access to the workplace. In addition, community members recruited participants and were compensated for this assistance at the suggestion of the staff.

## Challenges of Collaboration

The community-based staff came to this work with no research experience and limited general office skills. To address these challenges, their training included questionnaire administration, recruitment strategies, and skill-building training to allow them to manage the community office (effective communication; scheduling and record keeping; management; computer skills). The staff continue to develop these skills. The academic researchers came to the project with different limitations. Many of us had little prior experience with this community and were naïve about the lives of women in this rural area 130 miles from our universities. Like our community partners, we continue to learn.

Differences in communication, planning, and time management emphasized the cultural divide. Community-based staff correctly described the inability of participants to plan weeks or even days in advance for study appointments. This necessitated creating flexible office hours for exam times with study nurses. Even without the cultural differences between white academic researchers and black rural community members, the distance between the study site and institution limits daily interactions and makes the weekly meetings with the project manager an essential link.

The protection of the privacy of research participants takes on added dimensions within the context of both occupational health and community-based research (McPhaul and Lipscomb 2005). The planning, early participatory training, and ongoing conduct of the work have all revolved around maintaining privacy of participants, and the community staff are sensitive to these issues. Interviews conducted for exposure assessment and the ethnographic work took place in workers’ homes, private rooms in restaurants, friends’ houses, motel rooms, and our community office, always at the discretion of the woman being interviewed; they were recorded using aliases to protect privacy. Ongoing team discussions reinforce the importance of not revealing that any individual is a study participant in community encounters or in attempts to locate women for follow-up visits, as well as maintaining the confidence of all information shared in the data collection process.

We are asking workers to participate in a demanding protocol over several years. Both academic and community partners agreed that workers should be compensated for their time. Participants receive $40 for each data collection point involving an hour and a half interview and examination. Concern about effects of employment on health varies among participants, so compensation demonstrates respect for their time and their contribution to an effort for which they may not receive other direct benefits.

Both the academic and community staff are affected by the difficult lives of the poultry workers. We are saddened when participants must immediately use their incentive to purchase food for their children or kerosene to heat their trailer. We are frustrated by the complexities of occupational health concerns, especially when participants choose not to seek medical care for fear of losing their jobs or being identified as patients. We do not know if these fears are justified, but workers perceive these as real possibilities and consequently they influence behaviors.

The research process, including developing working relationships, building skills, recruiting workers, and maintaining their participation in this longitudinal study, has been time consuming. Recruitment, although designed to occur over time, has taken longer than initially planned. In economically depressed areas the immediate need for jobs can outweigh concerns about long-term health effects in the eyes of the community at large and in the eyes of workers. Low-wage industries such as poultry processing depend on a supply of unskilled labor; consequently, the industries have a vested interest in keeping economic growth low (Griffith 1993). Anxiety about job availability in the study area has been compounded by a poultry plant closure in August 2003. We believe the tension between health effects of employment and job concerns made the recruitment process more challenging, and it will likely affect perceptions about community outreach and education efforts.

Severance of the original community subcontract occurred at a pivotal point shortly before we were to begin enrollment of women in the longitudinal component of the project. The community staff members, who had participated in the early exposure interviews with workers and months of training for their project roles, maintained their commitment to the project. They immediately focused on the logistics of the work, conducting regular staff meetings and the early baseline interviews with participants in their own homes and by finding space for the project office.

## Rewards of Collaboration

There are significant rewards from our community-academic collaboration despite the challenges. The most notable achievement to date is the recruitment and retention of a cohort of 291 women, which would not have been possible without the community-based staff. Over 85% (87–97%) of those who were working at each follow-up period have remained in the study; 162 women (55.7%) remain in the cohort being followed. Data collection continues, with some participants having completed their seventh follow-up visit. Black women have not been adequately represented in epidemiologic studies, particularly studies related to occupational health, despite their high participation in the labor force and their higher-than-average levels of morbidity and mortality (Dennis and Neese 2000). Particularly in the face of a demanding protocol, a paternalistic employer, lack of alternative jobs, and the rural environment, this level of participation is significant.

Low-income black women are often labeled a “hard-to-reach” population by researchers (Wyatt et al. 2003). Barriers to their recruitment and retention in research studies are significant and include lack of transportation, costs, burdensome procedures, competing family responsibilities, lack of awareness, and distrust of investigators (Brown et al. 2000; Sengupta et al. 2000). Community-based staff intuitively deal with such issues. They have a thorough knowledge of the community, they understand the demands on working women, and they actively sought methods to accommodate the participants. Consistent with other community-based work (Corbie-Smith et al. 2003), there are indicators of trust associated with the project, not only as a safe place to participate in the research project, but also as a resource for other concerns. Women have come to the office to request a blood pressure check or have sought out the study nurses for information about pregnancy and delivery, for example.

The quality of data collection from our lengthy and complex questionnaire would not have been possible without in-person interviews. Our early analyses provide indicators of good face validity of the data. The musculoskeletal symptom reports are consistent with the hand-intensive nature of poultry-processing work and differ from those of other predominantly female occupational groups with different types of work exposures (Daraiseh et al. 2003). The distribution of upper extremity function scale measures among those with hand pain is similar to reports of Pransky et al. (1997) among employed clinic patients with upper-extremity disorders. These findings are consistent with successful reports of the use of workers to collect health outcome and exposure information from their peers (Dement et al. 2003; Lipscomb et al. 2003a, 2003b) and add to the evidence that community members can be successful in circumstances where academic researchers might not be (Avery et al. 2004).

Using descriptions from the interviews with the early key informants, a series of Likert-scaled questionnaire items were developed for behaviors that we believe represent plant-specific assertiveness. The interview data allowed us to frame the items using the women’s own words such as, “How likely are you to tell your line leader to ‘back off’?” Using factor analytic techniques (DeVellis 1991), we have identified a group of 11 items with good scale properties (Cronbach’s alpha 80.0). This will allow us to explore whether these behaviors are associated with longer tenure in the plant, their relationship to symptom development, and whether women with higher scores on the scale are more likely to alter the physical exposures of interest and thus introduce exposure variability that might otherwise be unrecognized. The approach we took was out of necessity, yet we would have been unlikely to identify this potentially important construct using standard methods of ergonomic assessment such as direct observation or videotape and review of several work cycles for each job.

The involvement of other community members in multiple facets of the study is conducive to outreach and community education. Formative work provided initial contacts with members of the community. While these women provided us with information about their work, we were able to provide them with information about the research effort. The same is true of women we interviewed for the life history portion of the study.

## Discussion

Community-based participatory research (CBPR) encompasses a wide range of research methods, with varying levels of involvement from communities and researchers. Guidelines for this approach have been described for work with communities in general (Green 2004; Metzler et al. 2003; Minkler 2004) and more specifically related to occupational and environmental health (Arcury et al. 2000; Mergler 1987; O’Fallon and Deary 2002). In actual practice, CBPR has depth and variation, and the SHOWW project has variable shades or dimensions of this approach (Israel et al. 1994, 1998; Sullivan 2001).

In contrast to situations in which academic investigators seek to study a population defined by their own research interests, this collaboration developed as academic researchers responded to a request of women in rural North Carolina to document health effects of work in poultry processing, the largest employer in their area. As such, the project provides an example of truly community-driven research.

The academic team viewed the request to some extent as a request for technical assistance. Early decisions about research design were made by the academic team based on input from women in the community, including the feasibility of the longitudinal design. Regardless of access, the fear and distrust of the industry among the worker community made the industry an unsuitable partner, and given this constraint, the research required community collaboration from the outset.

Equitable participation and influence between researchers and community is another tenet of CBPR. We believe this is a lofty goal to strive for but also one difficult to measure and seldom realized (Buchanan 1996; Wallerstein 1992). Our community partners are our local staff; they do not control funding or share equal power with the academic principal investigator. Nonetheless, within the confines of this structure, the staff have a strong voice in the research.

Responsibility was central to the decision to break ties with the original community partner. However, the change altered power structures and the decision was not made lightly. We had a responsibility to the poultry workers to find a model in which we could conduct the work, and we also felt responsible to the community staff who had become our active partners. In the model under which we continue our project, the staff do not work under the umbrella of a community organization or labor union but rather as employees of the university. As such, they do not have a separate organization from which to draw power, which at least theoretically, would give them strength and independence within the project. However, the community-based staff were actively involved in this decision. They openly reported and quickly demonstrated empowerment from the break, and they believe the independent project office has provided added legitimacy and recognition.

The restricted ability of many small community-based organizations to provide benefits of employment is often ignored in discussions of power and equity in community-based research. Health and life insurance, retirement contributions, and even paid vacation and sick leave are expected standards for university staff. The implications increase as the time committed by community staff to the project increases. In this project the community-based staff manage the project office, and their time commitments are greater than those of any other members of the academic team (30–35 hr per week). As university employees, they are eligible for corresponding benefits. These benefits, possible through our large university risk pool and the support afforded by substantial benefit rates on government grants, are often prohibitively expensive for small nonprofit and community organizations.

One could argue that failure to acknowledge these issues and realistically evaluate what is gained by not working under the perceived power structure of a community agency contributes to the very disparities in health we seek to address in our collaborative research. Is it equitable for university researchers not only to enjoy but to expect these benefits, yet deny them to our community colleagues because it is not the community standard? In attempts to guarantee substantial community involvement and leveling of the playing field between community and academic teams, proscribing the proportions of funds that must be designated to community organizations in CBPR projects may not have the desired result as the research is operationalized. The issues are not easy ones to address, but they cut to very nature of this collaboration focused on the health of working women.

We recognize that our project has both limitations and strengths. Occupational health research conducted without industry cooperation has merits and challenges. The volunteer cohort constitutes neither a full enumeration of potential workers nor a random sample of the population at risk. This will be a significant limitation of cross-sectional analyses of baseline findings in which subjects ideally would be selected based on exposure or disease. However, the longitudinal analyses focused on internal comparisons reduce the potential for bias. This does not negate the possibility of a healthy worker survivor effect (Checkoway et al. 2004), although it is diminished by successful follow-up of participants and the inclusion of a substantial number of women who were new to the industry.

The design we chose, which recruits and follows employed women, does not have an unexposed group. Unemployed women of working age may have characteristics that could confound results. They may belong to a different socioeconomic group, may not need to work, or may already have a work disability. Collecting detailed exposure assessment was not feasible for many work sites, so we focused our work only on women employed in poultry processing and made a conscious decision to trade external validity for improved internal validity.

Lack of access to the workplace constrains our ability to observe and directly measure exposures. Instead, we are using a multidimensional, indirect approach to exposure assessment. Previous comparative studies have shown that this approach, using qualitative or semiquantitative exposure assessments by workers and health and safety professionals, can provide a valid ranking of exposure levels relative to quantitative measurements (Flynn et al. 1991). In our case the development of the JEM also provides a method for assigning exposures that occurred before our observation period. This is particularly important in understanding cumulative exposures for the longer-term workers in our study. This process also engaged community women early in the project and improved the cultural relevance of questionnaire items.

Through the ethnographic life history interviews, women gave us insight into complex and subtle processes that may contribute to disparities in health. The women’s life stories revealed the effects that poorly funded schools, de facto segregation, teen childbearing, inadequate health care, cultural norms for work expectations, and a declining industrial base in the region may have on creating and maintaining disparities. Jointly, as academic researchers and representatives of the community, we intend to reach out to the community of poultry workers, the wider black and white communities, and academic, medical, and health policy circles. We have already found in sharing information from this project that the mix of quantitative epidemiologic data and qualitative ethnographic reports appeals to a wide variety of audiences.

We present our project as an example of how one collaboration developed with the calculated tradeoffs in design and logistics as challenges were faced. The model for community collaboration is not the one originally planned, but we do believe it allowed a modicum of success to date that would not have been realized otherwise. Research projects evolve as they move from plans on paper to actual work. Community collaborations add another dimension that we believe requires substantial flexibility to allow each to be effective under unique and unpredictable circumstances. We are not proposing that our model is ideal but rather one through which we have worked effectively while more equitably sharing benefits and resources.

In conclusion, in this research we focus on the physical environment of the workplace and the social environment, which includes the organization of work. However, it is important to see the workplace and the workers in the poultry plants as part of the larger economic and social environment. Working conditions—and occupational hazards—also vary by race and gender in ways that could affect worker health. Our research must be viewed within the broad context of economic realities of North Carolina, the United States, and the world. The migration of low-wage industry to economically disadvantaged areas of the rural South has led to the placement of poultry-processing plants in areas with a disproportionate share of women of color, many of whom are single heads of household with few economic alternatives.

Because “the discovery of hazards and proposed remedies have the potential to adversely affect the profit margin of business (Murray 2003, p 223),” there has been longstanding neglect for occupational safety and health that is not limited to this community or industry. Neither the academic research team nor the community collaborators think there are easy solutions, and we also recognize that the solutions do not lie solely in this community. The health of workers is influenced not just by work exposures but also by a complicated web in which government policy, racial history, geographic variation, economic opportunity, and longstanding patterns of exploitation may contribute to existing conditions and disparities in health among poor working women. The complexities of conducting occupational health research in this context, which places low-wage and nonwhite workers at risk of dangerous exposures and work conditions while at the same time diminishing their power and the ability of researchers to access the points of exposure effectively, requires creative designs and methods. Despite the challenges, we see the potential to document health conditions, gain a better understanding of the complex processes that influence them, and begin steps toward change through collaboration of community and academic researchers.

## Figures and Tables

**Table 1:** Key variables based on conceptual framework—SHOWW project.

	Variables	Measures used/source
Outcomes or responses	Musculoskeletal symptoms by body region	Modified from NIOSH symptom report items (NIOSH 2000) Hand diagram (Katz et al. 1990)
	Signs from physical exam	Modified from SHARP physical exam protocol (Viikari-Juntura 2000)
	MSDs	Combinations of signs and symptoms used to define working case definitions (Sluiter et al. 2001; Palmer et al. 2000 and Walker-Bone et al. 2002; Gerr et al. 2002)
	Acute work-related injury	Self-report
	Health-related quality of life^a^	SF-12 (Ware et al. 1996)
	Upper extremity function^a^	Upper extremity function scale (Pransky et al. 1997)
	Depressive symptoms^a^	CES-D (Radloff 1977)
Exposures	Work requirements Repetition, posture, force, temperature, tool use	Key informant interviews, project-specific self-report exposure tool
Modifiers	Work organization (decision latitude, control, demand, social support, job satisfaction)	Job Content Questionnaire (Karasek et al. 1998)
	Discrimination and response	Self-reported by race or gender and usual response (Krieger and Sidney 1996)
	Assertiveness at work	Scale measure from self-reported items; developed from key. informant interviews
	Coping style	John Henryism Active Coping Scale (James et al. 1987)
	Socioeconomic strain	Self-report of “Weeks you could be out of work without pay before loss of income would be a major problem.”
	Other health conditions	Medical history (select items based on possible relationship to MSDs—pregnancy, hormonal therapies, diabetes, etc.)

Abbreviations: CES-D, Center for Epidemiologic Studies Depression Scale; SF-12, SF-12 health survey; SHARP, Safety and Health Assessment and Research Program.

a Outcome of interest and potential modifier.

**Table 2:** Community contributions to SHOWW study and support provided to community-based staff.

**Community members**
Initiated request for academic assistance
Influenced decision to circumvent industry involvement
Influenced decision to conduct longitudinal study
Informed exposure assessment and other data collection tools
Participate in longitudinal research
Participate in ethnographic interviews
Recruit eligible participants
**Community members as paid staff/collaborators**
Provide valuable insight and helpful strategies for working with community
Participate in key decision making
Influenced decision to include ethnographic work
Arranged key informant interviews early in study development process
Selected location and site for community office
Recruit and retain participants using social networks and “snowball”method of recruitment
Manage daily office activities including recruitment incentives and advertising
Coordinate office schedules with study nurses
Transport participants; provide childcare
Participate in local community outreach and education
Provide outreach and education to academic community
**Support provided to community-based staff**
Participatory training—study design, recruitment/retention methods, interviewing skills, protection of participants’ confidentiality
Weekly staff meetings with project manager
Topic specific training
Office management (record keeping, database management)
MSDs
Principles of ergonomics
Daily availability by phone/e-mail of university team member as resource; study physician always available by pager
University benefits of employment—health and life insurance, paid vacation and sick leave, etc.
